# Geometry of Statistical Manifolds

**DOI:** 10.3390/e27111110

**Published:** 2025-10-27

**Authors:** Paul W. Vos

**Affiliations:** Department of Public Health, Brody School of Medicine, East Carolina University, Greenville, NC 27834, USA; vosp@ecu.edu

**Keywords:** Hilbert bundle, generalized estimation, slope, information, parameter-invariance, nuisance parameters, orthogonalization

## Abstract

A statistical manifold *M* can be defined as a Riemannian manifold each of whose points is a probability distribution on the same support. In fact, statistical manifolds possess a richer geometric structure beyond the Fisher information metric defined on the tangent bundle TM. Recognizing that points in *M* are distributions and not just generic points in a manifold, TM can be extended to a Hilbert bundle HM. This extension proves fundamental when we generalize the classical notion of a point estimate—a single point in *M*—to a function on *M* that characterizes the relationship between observed data and each distribution in *M*. The log likelihood and score functions are important examples of generalized estimators. In terms of a parameterization $\theta :M\to \Theta \subset R^{k}$, $\hat{\theta }$ is a distribution on Θ while its generalization $g_{\hat{\theta }}=\hat{\theta }-E\hat{\theta }$ as an *estimate* is a function over Θ that indicates inconsistency between the model and data. As an *estimator*, $g_{\hat{\theta }}$ is a distribution of functions. Geometric properties of these functions describe statistical properties of $g_{\hat{\theta }}$. In particular, the expected slopes of $g_{\hat{\theta }}$ are used to define $\Lambda (g_{\hat{\theta }})$, the Λ-information of $g_{\hat{\theta }}$. The Fisher information *I* is an upper bound for the Λ-information: for all *g*, Λ(g)≤I. We demonstrate the utility of this geometric perspective using the two-sample problem.

## 1. Introduction

Statistical manifolds provide a geometric framework for understanding families of probability distributions. While traditionally defined as Riemannian manifolds equipped with the Fisher information metric, their structure extends beyond this basic framework. Lauritzen [1] identified an additional skewness tensor, and Amari [2] also noticed this additional structure which he used to define a family of connections including both the metric connection and a dual pair—the mixture and exponential connections. This duality, first observed by Efron [3], reveals geometric structure beyond the Riemannian setting, though this previous work remained confined to the tangent bundle.

Amari [4] introduced a Hilbert space extension of the tangent bundle which Amari and Kumon [5] applied to estimating functions. Kass and Vos (Section 10.3) [6] also describe statistical Hilbert bundles which Pistone [7] extends to other statistical bundles in the nonparametric setting where extra care is required when the sample space is not finite. Recent developments have expanded the geometric perspective on the role of the Hilbert bundle in parametric inference when the traditional approach to statistical inference is replaced with Fisher’s view of estimation.

Classical statistical inference separates estimation and hypothesis testing into distinct frameworks. Point estimators map from the sample space to the parameter space, with their local properties described through the tangent bundle. Test statistics similarly rely on tangent bundle geometry. The log likelihood and its derivative, the score function, bridge these approaches by providing both estimation methods (maximum likelihood) and testing procedures (likelihood ratio and score tests). Godambe [8] extended the score’s role in estimation through estimating equations, yet the fundamental separation between testing and estimation persisted.

Building on Fisher’s [9] conception of estimation as a continuum of hypothesis tests, Vos [10] unified these approaches by replacing point estimators with generalized estimators—functions on the parameter space that geometrically represent surfaces over the manifold. These generalized estimators shift the inferential focus from individual parameter values to entire functions, whose properties are naturally characterized within the Hilbert bundle framework.

This paper demonstrates the advantages of generalized estimators and the utility of the Hilbert bundle perspective specifically for the two-sample problem. We show how the orthogonalized score achieves information bounds as a consequence of its membership in the tangent bundle, while other generalized estimators, residing only in the larger Hilbert bundle, suffer information loss measured by their angular deviation from the tangent space.

## 2. Statistical Manifolds

Let $M^{X}$ be a family of probability measures with common support X. While X can be an abstract space, for most applications, $X\subset R^{d}$. Each point in $M^{X}$ represents a candidate model for a population whose individuals take values in X.

We consider inference based on a sample denoted by *y*, with corresponding sample space Y. The relationship between X and Y depends on three factors: the sampling plan, any conditioning applied, and dimension reduction through sufficient statistics. In the simplest case—a simple random sample of size *n* without conditioning or dimension reduction—we have $Y=X^{n}$.

Let $M=M^{Y}$ denote the family of probability measures on Y induced by $M^{X}$ through the sampling plan. For the simple random sampling case:

$$M=\left\{m:m\left(y\right)=\prod_{i=1}^{n}m^{X}\left(x_{i}\right),\;m^{X}\in M^{X}\right\}.$$

For any real-valued measurable function *h*, we define its expected value at m∈M as

$$E_{m}h=\left\{\begin{array}{cc} \int_{Y}h\left(y\right)m\left(y\right)d\mu & \mathrm{when}\;Y\;\mathrm{is}\;\mathrm{continuous}\\ \sum_{y\in Y}h\left(y\right)m\left(y\right) & \mathrm{when}\;Y\;\mathrm{is}\;\mathrm{discrete} \end{array}\right.$$

The Hilbert space associated with *M* consists of all square-integrable functions:

$$H_{M}=\left\{h:E_{m}h^{2}<\infty ,\;\forall \;m\in M\right\}.$$

This space carries a family of inner products indexed by points in *M*:

$${\langle h,h^{\prime }\rangle }_{m}=E_{m}\left(hh^{\prime }\right)\;\mathrm{for}\;\mathrm{all}\;h,h^{\prime }\in H_{M}.$$

When ${\langle h,h^{\prime }\rangle }_{m}=0$, we say that *h* and $h^{\prime }$ are *m-orthogonal* and write $h{⊥}_{m}h^{\prime }$.

We construct the Hilbert bundle over *M* by associating a copy of $H_{M}$ to each point:

$$H\;M=M\times H_{M}.$$

The fiber at *m*, denoted $H_{m}$ or $H_{m}M$, inherits the inner product ${\langle \cdot ,\cdot \rangle }_{m}$. For inference purposes, we decompose each fiber into the space of constant functions and its orthogonal complement:

(1) $$H_{m}=H_{m}^{⊥}⊕H_{m}^{0}\;\mathrm{where}\;H_{m}^{⊥}{⊥}_{m}H_{m}^{0}.$$

Here, $H_{m}^{⊥}=\left\{h\in H_{m}:E_{m}h=0\right\}$ consists of centered functions, while $H_{m}^{0}$ contains the constants. Note that $E_{m}h={\langle h,1\rangle }_{m}$ and $H_{m}^{0}$ is independent of *m*. As decomposition (1) holds fiberwise, we obtain a global decomposition:

(2) $$H\;M=H^{⊥}\;M⊕H^{0}M\;\mathrm{where}\;H^{⊥}M⊥H^{0}M.$$

The bundle HM extends the tangent bundle TM, which emerges naturally through parameterization. We assume that *M* admits a global parameterization—while not strictly necessary, this simplifies our exposition by avoiding coordinate charts. We require this parameterization to be a diffeomorphism.

Consider a parameterization $\theta :M\to \Theta \subset R^{d}$ with inverse $\theta^{-1}:\Theta \to M$. For a specific distribution $m_{\circ }\in M$, we write $\theta_{\circ }=\theta \left(m_{\circ }\right)$ for its parameter value. When considering all distributions simultaneously, we write θ=θ(m), where context distinguishes between θ as a point in Θ (left side) and as a function (right side).

For notational convenience, we denote the distribution corresponding to parameter value $\theta_{\circ }$ as $m_{\theta_{\circ }}=\theta^{-1}\left(\theta_{\circ }\right)$. This allows us to write the following:

$$m_{\theta }=\theta^{-1}\left(\theta \right)$$

where, again, context clarifies whether $m_{\theta }$ refers to the function $\theta^{-1}$ or its value.

With this parameterization, the Hilbert bundle can be expressed as

$$H\;M=\Theta \times H_{M}$$

allowing us to index fibers by parameter values: $H_{\theta }M=H_{m_{\theta }}M$.

The log likelihood function plays a fundamental role in our geometric framework. On *M*, it is the function $\ell_{M}:Y\times M\to R$ defined by $\ell_{M}\left(y,m\right)=\log m\left(y\right)$. Through the parameterization, this induces $\ell_{\Theta }:Y\times \Theta \to R$ given by $\ell_{\Theta }\left(y,\theta \right)=\log m_{\theta }\left(y\right)$. When the parameterization is clear from context, we simply write *ℓ* for $\ell_{\Theta }$.

The partial derivatives of *ℓ* with respect to the parameters,

$$\left\{\frac{\partial \ell }{\partial \theta^{1}},\frac{\partial \ell }{\partial \theta^{2}},\ldots ,\frac{\partial \ell }{\partial \theta^{d}}\right\},$$

evaluated at $\theta_{\circ }$, form a basis for the tangent space $T_{\theta_{\circ }}M$. For all i=1,…,d and all m∈M, $0<E_{m}{\left(\partial \ell /\partial \theta^{i}\right)}^{2}<\infty$, ensuring that TM⊂HM. In fact, $T\;M\subset H^{⊥}M$ as $E_{m}\left(\partial \ell /\partial \theta^{i}\right)$ vanishes on *M*.

## 3. Functions on M

The log likelihood function *ℓ* and its derivatives are central to statistical inference. Traditionally, these serve as tools to find point estimates—particularly the maximum likelihood estimate (MLE)—and to characterize the estimator’s properties. We adopt a different perspective: we treat *ℓ* and its derivatives as primary inferential objects rather than mere computational tools. This approach aligns with Fisher’s conception of estimation as a continuum of tests.

As the log likelihood ratio for comparing models with parameters $\theta_{1}$ and $\theta_{2}$ is the difference $\log m_{\theta_{1}}\left(y\right)-\log m_{\theta_{2}}\left(y\right)$ and adding an arbitrary constant to each term does not affect this difference, we define the log likelihood so that ℓ≤0 for each fixed *y*. Thus we work with

$$\ell \left(y,\theta \right)=\log m_{\theta }\left(y\right)-\underset{m\in M}{\sup}\log m\left(y\right).$$

As an inferential function, |ℓ(y,θ)| quantifies the dissonance between observation *y* and distribution $m_{\theta }$. While the MLE set {θ:ℓ(y,θ)=0} identifies parameters with minimal dissonance, our emphasis shifts to characterizing the full landscape of dissonance across the manifold. While “dissonance” lacks a precise mathematical definition, it can be thought of as the evidence in *y* against the model at θ—essentially, a test statistic evaluated at *y* for the null hypothesis specifying $m_{\theta }$. We use the notation $\hat{\theta }$ for the MLE when it is unique. When the MLE set is empty we say that the MLE does not exist. Note that *ℓ* is defined even when the MLE does not exist or is not unique; the only requirement is that $\sup_{m}\log m\left(y\right)<\infty$.

The log likelihood exemplifies a broader class of *generalized estimators*: functions G:Y×Θ→R where, for almost every y∈Y, the function G(y,·) measures dissonance between *y* and distributions across *M*. Like *ℓ*, we can normalize *G* so that G≤0.

Consider the geometric interpretation. For a function f:Θ→R, let ℷ(f)={(θ,f(θ)):θ∈Θ}⊂Θ×R denote its graph. The graphs ℷ(−ℓ(y,·)) and ℷ(−G(y,·)) form *d*-dimensional surfaces over $\Theta \subset R^{d}$. We compare these surfaces through their gradients:

s(y,θ)=∇ℓ(y,θ)andg(y,θ)=∇G(y,θ)

where $\nabla ={\left(\partial /\partial \theta^{1},\ldots ,\partial /\partial \theta^{d}\right)}^{t}$.

Viewing these as estimators requires replacing the fixed observation *y* with the random variable *Y*. Then ℷ(−ℓ(Y,·)) and ℷ(−G(Y,·)) become random surfaces, while s(Y,θ) and g(Y,θ) become random gradient fields. The score components ${\left\{s^{i}\left(Y,\theta \right)\right\}}_{i=1}^{d}$ span the tangent space: $\mathrm{span}\left\{s\left(Y,\theta \right)\right\}=T_{\theta }M$. The key difference between generalized estimation described in this paper and the estimating equations of Godambe and Thompson [11] lies in their inferential approach: the former focuses on the distribution of graph slopes (gradients in a linear space), while the latter examines the distribution of where graphs intersect the horizontal axis (roots of *g*).

Under mild regularity conditions, the components of g(Y,θ) span a subspace of $H_{\theta }M$ of dimension *d* although generally not $T_{\theta }M$. Strictly speaking, span{s(Y,θ)} is isomorphic rather than equal to $T_{\theta }M$, as the former consists of vectors attached to the surface ℷ(−ℓ(y,·)) while the latter are attached to *M* (equivalently, to Θ). As shown in Vos and Wu [12], this precise relationship between the log likelihood surface and the manifold ensures that score-based estimators attain the information bound.

This perspective fundamentally shifts our focus. Rather than comparing point estimators through their variance or mean squared error on the parameter space Θ, we compare the linear spaces spanned by the components of generalized estimators within the Hilbert bundle HM.

For point estimator $\overset{ˇ}{\theta }$, define its associated generalized estimator:

$$g_{\overset{ˇ}{\theta }}=\overset{ˇ}{\theta }-E\overset{ˇ}{\theta }.$$

The estimator must have nonzero variance, $0<V(\overset{ˇ}{\theta })<\infty$ for all θ∈Θ, so that $\overset{ˇ}{\theta }\in H_{M}$. Instead of traditional comparisons between $\hat{\theta }$ (the MLE) and $\overset{ˇ}{\theta }$, we compare the spaces spanned by s=s(Y,θ) and $g_{\overset{ˇ}{\theta }}=g_{\overset{ˇ}{\theta }}\left(Y,\theta \right)$ through their Λ-information—a generalization of Fisher information to arbitrary generalized estimators. Geometrically, the relationship between *s* and $g_{\overset{ˇ}{\theta }}$ is characterized by angles between their component vectors. Statistically, this translates to correlations between the corresponding random variables. The Λ-information is defined by the left-hand side of Equation (13) which also shows the role of the correlation.

Generalized estimators offer particular advantages when nuisance parameters are present. For point estimators, one seeks a parameterization where nuisance and interest parameters are orthogonal—a goal not always achievable. When working in HM rather than Θ, orthogonalization remains important but becomes more flexible: the choice of nuisance parameterization becomes immaterial as orthogonalization occurs within HM itself.

The information bound for the interest parameter is attained by restricting generalized estimators to be orthogonal to the nuisance parameter’s score components. The general framework is developed in Vos and Wu [12]; we illustrate the approach through the special case for comparing two populations in the following section.

## 4. Comparing Two Populations

We now develop the general framework for comparing two distributions from the same parametric family. The next section applies this framework to the more specific case of 2×2 contingency tables.

Let $M^{X}$ be a one-parameter family of distributions on X⊂R, and let $M^{Y}$ be the corresponding family of sampling distributions on Y. While we work primarily with sampling distributions in $M^{Y}$, we use superscripts to distinguish when necessary: $m^{Y}$ denotes a sampling distribution obtained from population distribution $m^{X}$.

For simple random sampling outside exponential families, $Y=X^{n}$. Within exponential families, Y represents the support of the sufficient statistic. For example, when $M^{X}$ consists of Bernoulli distributions with success probability p∈(0,1), the family $M^{Y}$ consists of binomial distributions for *n* trials with sample space Y={0,1,2,…,n}.

Let $\theta_{Y}:M^{Y}\to \Theta \subset R$ parameterize $M^{Y}$. We define the population parameterization $\theta_{X}$ to ensure consistency: $\theta_{Y}^{-1}\circ \theta_{X}\left(m^{X}\right)=m^{Y}$. Thus, each parameter value simultaneously labels both a population distribution in $M^{X}$ and its corresponding sampling distribution in $M^{Y}$. As our focus is on sampling distributions, we simplify notation by dropping the subscript $\theta =\theta_{Y}$.

The score function for parameter θ is

$$\ell_{/\theta }=\frac{\partial \ell }{\partial \theta }=\alpha Z$$

where we factor the score into its magnitude $\alpha =\sqrt{V(\ell_{/\theta })}$ and its standardized version *Z* with EZ=0 and V(Z)=1. Both α and *Z* depend on θ and, thus, vary across $M^{Y}$.

Under reparameterization ξ of θ, the standardized score *Z* remains invariant while the coefficient transforms as α∂θ/∂ξ. The coefficient α equals the square root of the total Fisher information: $\alpha =\sqrt{I_{\theta }}=\sqrt{ni_{\theta }}$ where $i_{\theta }$ is the Fisher information per observation. For the binomial family, $Z=\left(Y-np\right)/\sqrt{np(1-p)}$.

Now consider independent samples of sizes $n_{1}$ and $n_{2}$ from two distributions in $M^{X}$. The manifold of joint population distributions is

$$M^{X\times X}=\left\{m=m_{1}m_{2}:m_{1},m_{2}\in M^{X}\right\}$$

with corresponding manifold of joint sampling distributions:

$$M^{Y_{1}\times Y_{2}}=\left\{m=m_{1}m_{2}:m_{1}\in M^{Y_{1}},m_{2}\in M^{Y_{2}}\right\}.$$

The parameterization $\theta_{X}$ of $M^{X}$ induces natural parameterizations $\theta_{X\times X}={\left(\theta_{X},\theta_{X}\right)}^{t}$ on $M^{X\times X}$ and $\theta_{Y_{1}\times Y_{2}}={\left(\theta_{Y_{1}},\theta_{Y_{2}}\right)}^{t}$ on $M^{Y_{1}\times Y_{2}}$. These share the same image $\Theta_{1}\times \Theta_{2}$ where $\Theta_{1}=\Theta_{2}=\theta_{X}\left(M^{X}\right)$. Setting $M=M^{Y_{1}\times Y_{2}}$ and $\theta ={\left(\theta_{1},\theta_{2}\right)}^{t}$, each point in Θ labels both a joint sampling distribution and its generating population distribution.

The hypothesis that both samples arise from the same distribution corresponds to the diagonal submanifold:

$$M_{diag}^{X\times X}=\left\{m=m_{1}m_{2}:m_{1}=m_{2}\in M^{X}\right\}$$

with parameter space:

$$\Theta_{diag}=\left\{{\left(\theta_{1},\theta_{2}\right)}^{t}\in \Theta :\theta_{1}=\theta_{2}\right\}.$$

The joint parameter $\theta ={\left(\theta_{1},\theta_{2}\right)}^{t}$ yields two score functions:

$$\ell_{/\theta_{1}}=\alpha_{1}Z_{1}\;\mathrm{and}\;\ell_{/\theta_{2}}=\alpha_{2}Z_{2}$$

where $Z_{1}$ and $Z_{2}$ are orthonormal at each m∈M:

$${\langle Z_{1},Z_{2}\rangle }_{m}=E_{m}\left(Z_{1}Z_{2}\right)=0.$$

To compare the distributions, we reparameterize using the difference $\delta =\theta_{1}-\theta_{2}$ as our interest parameter and $\tau =\theta_{1}+\theta_{2}$ as the nuisance parameter. The inverse transformation gives $\theta_{1}=\frac{1}{2}\left(\tau +\delta \right)$ and $\theta_{2}=\frac{1}{2}\left(\tau -\delta \right)$, yielding scores:

(3) $$\begin{array}{cc} \ell_{/\delta } & =\frac{\partial \theta_{1}}{\partial \delta }\ell_{/\theta_{1}}+\frac{\partial \theta_{2}}{\partial \delta }\ell_{/\theta_{2}}=\frac{1}{2}\alpha_{1}Z_{1}-\frac{1}{2}\alpha_{2}Z_{2} \end{array}$$

(4) $$\begin{array}{cc} \ell_{/\tau } & =\frac{\partial \theta_{1}}{\partial \tau }\ell_{/\theta_{1}}+\frac{\partial \theta_{2}}{\partial \tau }\ell_{/\theta_{2}}=\frac{1}{2}\alpha_{1}Z_{1}+\frac{1}{2}\alpha_{2}Z_{2}. \end{array}$$

Let $Z_{\nu }$ denote the unit vector in the direction of $\ell_{/\tau }$, satisfying $\langle Z_{\nu },\ell_{/\tau }\rangle >0$, $EZ_{\nu }=0$, and $|Z_{\nu }|=1$. As $Z_{\nu }=\ell_{/\tau }/\left|\ell_{/\tau }\right|$ remains invariant under monotonic reparameterizations of τ, we use the subscript ν (for nuisance). In terms of the basis $\left\{Z_{1},Z_{2}\right\}$:

$$Z_{\nu }=\frac{\alpha_{1}Z_{1}+\alpha_{2}Z_{2}}{\sqrt{\alpha_{1}^{2}+\alpha_{2}^{2}}}=\frac{I_{\theta_{1}}^{1/2}Z_{1}+I_{\theta_{2}}^{1/2}Z_{2}}{\sqrt{I_{\theta_{1}}+I_{\theta_{2}}}}.$$

Let *h* be a point estimator or test statistic for δ. The function *h* is a generalized pre-estimator provided h−Eh is a generalized estimator. For any pre-estimator *h* of δ, define its orthogonalized version:

$$h^{⊥}=\left(h-Eh\right)-\langle h,Z_{\nu }\rangle Z_{\nu }=\left|h\right|\left(Z_{h}-\rho_{h\nu }Z_{\nu }\right)$$

where $Z_{h}=\left(h-Eh\right)/\left|h\right|$ is the standardized direction and $\rho_{h\nu }=\langle Z_{h},Z_{\nu }\rangle$ is the correlation with the nuisance direction.

To ensure that inference is independent of the nuisance parameter, we work with orthogonalized generalized estimators $g=h^{⊥}$:

(5) $$\begin{array}{ccc} g & = & \left|h|(Z_{h}-\rho_{h\nu }Z_{\nu }\right)\\ & = & \left|g\right|Z_{g}=\sqrt{1-\rho_{h\nu }^{2}}\left|h\right|Z_{g}. \end{array}$$

When *h* is the score for δ, the orthogonalized score becomes

(6) $$s^{⊥}=\sqrt{I_{\delta }^{⊥}}Z_{s^{⊥}}=\sqrt{\left(1-\rho_{s\nu }^{2}\right)I_{\delta }}Z_{s^{⊥}}$$

where $I_{\delta }^{⊥}$ is the information after orthogonalization. The proportion of information loss due to the nuisance parameter is the square of the correlation between the interest and nuisance parameters

(7) $$\rho_{s\nu }^{2}=\frac{I_{\delta }-I_{\delta }^{⊥}}{I_{\delta }}.$$

This loss cannot be recovered by reparameterization. Geometrically, $\rho_{s\nu }=\cos∠\left(Z_{s},Z_{\nu }\right)$, so the proportional information loss equals the squared cosine of the angle between the score and the tangent space of $M_{\delta_{\circ }}=\delta^{-1}\left(\delta_{\circ }\right)$. The submanifold $M_{\delta_{\circ }}$ depends on the choice of interest parameter and is integral to the inference problem.

The orthogonalized Fisher information $I_{\delta }^{⊥}$ is additive on the reciprocal scale:

(8) $${\left(I_{\delta }^{⊥}\right)}^{-1}=I_{\theta_{1}}^{-1}+I_{\theta_{2}}^{-1}.$$

Equation (8) is established as follows. The orthogonalized score is a linear combination of the orthonormal basis vectors $Z_{1}$ and $Z_{2}$,

(9) $$\begin{array}{ccc} s^{⊥} & = & \ell_{/\delta }-\frac{\langle \ell_{/\delta },\ell_{/\tau }\rangle }{\langle \ell_{/\tau },\ell_{/\tau }\rangle }\ell_{/\tau }\\ & = & \left(\frac{1}{2}\alpha_{1}Z_{1}-\frac{1}{2}\alpha_{2}Z_{2}\right)-\left(\frac{\alpha_{1}^{2}-\alpha_{2}^{2}}{\alpha_{1}^{2}+\alpha_{2}^{2}}\right)\left(\frac{1}{2}\alpha_{1}Z_{1}+\frac{1}{2}\alpha_{2}Z_{2}\right)\\ & = & \frac{\alpha_{1}\alpha_{2}}{\alpha_{1}^{2}+\alpha_{2}^{2}}\left(\alpha_{2}Z_{1}-\alpha_{1}Z_{2}\right)\\ & = & \frac{\alpha_{1}\alpha_{2}}{\sqrt{\alpha_{1}^{2}+\alpha_{2}^{2}}}Z_{s^{⊥}}. \end{array}$$

As $|s^{⊥}{|}^{2}=I_{\delta }^{⊥}$ and $\alpha_{i}^{2}=I_{\theta_{i}}$:

(10) $$I_{\delta }^{⊥}=\frac{\alpha_{1}^{2}\alpha_{2}^{2}}{\alpha_{1}^{2}+\alpha_{2}^{2}}=\frac{I_{\theta_{1}}I_{\theta_{2}}}{I_{\theta_{1}}+I_{\theta_{2}}}$$

and taking the reciprocal of both sides of (10) gives (8). Substituting into (7) with $I_{\delta }=\frac{1}{4}\left(I_{\theta_{1}}+I_{\theta_{2}}\right)$ shows

$$\rho_{s\nu }^{2}=\frac{{\left(I_{\theta_{1}}-I_{\theta_{2}}\right)}^{2}}{I_{\theta_{1}}+I_{\theta_{2}}}$$

which means that the information loss due to the nuisance parameter is proportional to the squared difference in the Fisher information for the distributions being compared. Using Equation (9), the orthogonalized score in terms of the basis vectors $Z_{1}$ and $Z_{2}$ is

$$\begin{array}{cc} s^{⊥} & =I_{\delta }^{⊥}\left(\frac{Z_{1}}{\sqrt{I_{\theta_{1}}}}-\frac{Z_{2}}{\sqrt{I_{\theta_{2}}}}\right). \end{array}$$

The basis $\left\{Z_{s^{⊥}},Z_{\nu }\right\}$ is obtained from $\left\{Z_{1},Z_{2}\right\}$ using the linear transformation

(11) $$\left(\begin{array}{c} Z_{s^{⊥}}\\ Z_{\nu } \end{array}\right)=\frac{1}{\sqrt{\alpha_{1}^{2}+\alpha_{2}^{2}}}\left(\begin{array}{cc} \alpha_{2} & -\alpha_{1}\\ \alpha_{1} & \alpha_{2} \end{array}\right)\left(\begin{array}{c} Z_{1}\\ Z_{2} \end{array}\right)$$

which is a rotation through an angle of $\cos^{-1}\sqrt{I_{\theta_{2}}/\left(I_{\theta_{1}}+I_{\theta_{2}}\right)}$. When θ is a location parameter, $I_{\theta_{1}}$ and $I_{\theta_{2}}$ are constant on *M*. With equal sample sizes ($n_{1}=n_{2}$), the rotation angle is π/4 and $Z_{s^{⊥}}\propto \left(Z_{1}-Z_{2}\right)$.

While $Z_{s^{⊥}}\in T\;M$, for general estimators *g* we have $Z_{g}\in H\;M$ but $Z_{g}\notin T\;M$ unless $g=s^{⊥}$. This distinction explains why general estimators fail to achieve the information bound. The Λ-information of *g* is $\Lambda \left(g\right)=\rho_{gs^{⊥}}^{2}I_{\delta }^{⊥}$, where $\rho_{gs^{⊥}}$ is the correlation between $Z_{g}$ and $Z_{s^{⊥}}$.

The null hypothesis $H_{0}:\delta =0$ deserves special attention. While $H:\delta =\delta_{\circ }$ generally depends on the parameterization choice, $H_{0}:\delta =0$ is parameterization-invariant as it is equivalent to $H_{0}:\theta_{1}=\theta_{2}$. Under simple random sampling with $I_{\theta }=nI_{\theta }$, the standardized orthogonalized score on $M_{0}=\delta^{-1}\left(0\right)$ becomes

$$Z_{s^{⊥}}=\frac{n_{2}^{1/2}Z_{1}-n_{1}^{1/2}Z_{2}}{\sqrt{n_{1}+n_{2}}}$$

which is invariant across all parameterizations θ. This invariance does not hold for test statistics based on point estimators like ${\hat{\theta }}_{1}-{\hat{\theta }}_{2}$, whose form depends on whether we parameterize using proportions, log-proportions, or log-odds.

## 5. Comparing Two Bernoulli Distributions

We now specialize the general framework to comparing two Bernoulli distributions, establishing the geometric structure that underlies inference for 2×2 contingency tables.

For the Bernoulli sample space X={0,1}, the manifold of population distributions is

$$M^{X}=\left\{m:0<m\left(1\right)<1,m\left(0\right)+m\left(1\right)=1\right\}$$

with natural parameterization $p_{X}\left(m\right)=m\left(1\right)$. For a sample of size *n*, the sufficient statistic has support Y={0,1,…,n}, yielding the manifold of binomial sampling distributions:

MY=m:m(y)=nypy(1−p)n−y,0<p<1.

A natural bijection exists between $M^{X}$ and $M^{Y}$: each population distribution determines a unique sampling distribution. We define $p_{Y}$ to make this bijection $p_{Y}^{-1}\circ p_{X}$. Similarly, for any alternative parameterization $\theta_{X}$ (such as $\theta_{X}\left(m\right)=\log\left(m\left(1\right)/m\left(0\right)\right)$), we define $\theta_{Y}$ so that the bijection equals $\theta_{Y}^{-1}\circ \theta_{X}$.

For independent samples of sizes $n_{1}$ and $n_{2}$, the joint manifolds are

$$\begin{array}{cc} M^{X\times X} & =\{m=m_{1}m_{2}:m_{1},m_{2}\in M^{X}\}\\ M=M^{Y_{1}\times Y_{2}} & =\{m=m_{1}m_{2}:m_{1}\in M^{Y_{1}},m_{2}\in M^{Y_{2}}\}. \end{array}$$

Using the proportion parameterization $p={\left(p_{1},p_{2}\right)}^{t}\in {\left(0,1\right)}^{2}$, the sampling distribution at *p* is

m(y1,y2)=n1y1n2y2p1y1(1−p1)n1−y1p2y2(1−p2)n2−y2

for ${\left(y_{1},y_{2}\right)}^{t}\in Y_{1}\times Y_{2}$, with corresponding population distribution:

$$m_{X\times X}\left(x_{1},x_{2}\right)=p_{1}^{x_{1}}{\left(1-p_{1}\right)}^{1-x_{1}}p_{2}^{x_{2}}{\left(1-p_{2}\right)}^{1-x_{2}}$$

for ${\left(x_{1},x_{2}\right)}^{t}\in {\left\{0,1\right\}}^{2}$.

The Hilbert space for this manifold consists of all real-valued functions on the finite sample space:

(12) $$H_{M}=\left\{h:Y_{1}\times Y_{2}\to R:\sum_{y_{1},y_{2}}h^{2}\left(y_{1},y_{2}\right)m\left(y_{1},y_{2}\right)<\infty ,\forall m\in M\right\}.$$

As the support is finite, $H_{M}$ includes all finite-valued functions. The tangent space at *m* is the two-dimensional subspace:

$$T_{m}M=\mathrm{span}\left\{Y_{1}-n_{1}p_{1},Y_{2}-n_{2}p_{2}\right\}$$

where $p_{1}=p_{1}\left(m\right)$ and $p_{2}=p_{2}\left(m\right)$.

Table 1 summarizes the Fisher information per observation for three common parameterizations of the Bernoulli distribution, each offering different advantages for inference.

We illustrate our geometric framework using data from Mendenhall et al. [13], who conducted a retrospective analysis of laryngeal carcinoma treatment. Disease was controlled in 18 of 23 patients treated with surgery alone and 11 of 19 patients treated with irradiation alone ($y_{1}=18$, $n_{1}=23$, $y_{2}=11$, $n_{2}=19$). We use this data to compare the orthogonalized score $s^{⊥}$ with other generalized estimators when the interest parameter is the log odds ratio $\delta =\log\left(p_{1}/\left(1-p_{1}\right)\right)-\log\left(p_{2}/\left(1-p_{2}\right)\right)$.

### 5.1. Orthogonalized Score

The score has two key properties: at each point in the sample space $\ell_{/\delta }$ is a smooth function on the parameter space, and at each each point in the manifold $\ell_{/\delta }$ is a distribution on the sample space. Formally, for *y* fixed $\ell_{/\delta }=\ell_{/\delta }\left(y,\cdot \right)\in C^{1}\left(\Delta \right)$ and for δ fixed $\ell_{/\delta }=\ell_{/\delta }\left(\cdot ,\delta \right)\in H_{m}M$ when there is no nuisance parameter. As δ is the interest parameter we use the notation *s* for the score $\ell_{/\delta }$. These properties persist after orthogonalization and standardization to obtain $s^{⊥}$ and $Z_{s^{⊥}}$.

**Figure 1:**
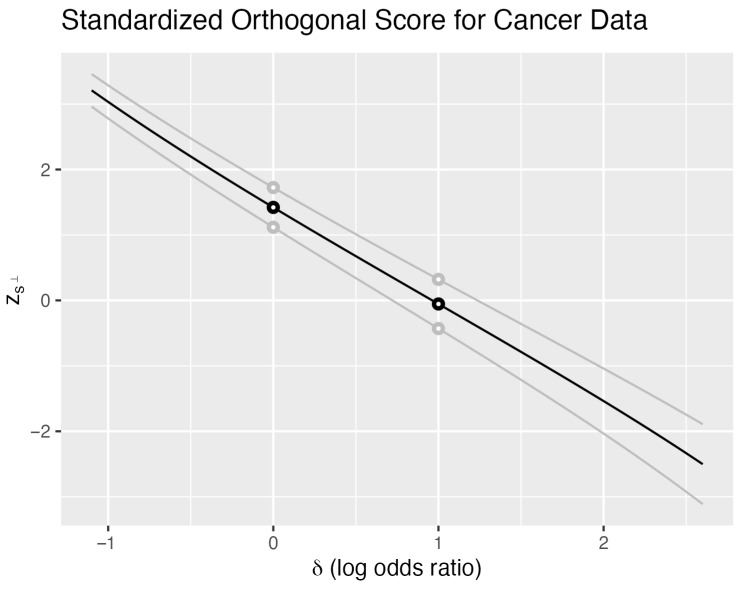
Cancer data—orthogonalized score. Standardized orthogonal score $z_{s^{\;⊥}}$ as a function of the log odds difference δ. The observed data (18 of 23 surgery successes, 11 of 19 irradiation successes) yields the black curve. Two additional sample points shown in gray illustrate the family of 480 possible curves. The nuisance parameter is fixed at $\xi =n_{1}p_{1}+n_{2}p_{2}=29$. Horizontal lines at z=±2 intersect the observed curve to yield an approximate 95% confidence interval for δ.

Figure 1 illustrates these properties for $Z_{s^{⊥}}$ using the cancer data. The black curve shows $Z_{s^{\;⊥}}$ evaluated at the observed sample $\left(y_{1}=18,y_{2}=11\right)$ as a function of δ, with the nuisance parameter fixed at $\xi_{\circ }=29$. Each of the 480 points in the sample space $Y_{1}\times Y_{2}$ generates such a curve; two additional examples appear in gray. We distinguish the family of curves $Z_{s^{⊥}}$ (uppercase) from the specific observed curve $z_{s^{⊥}}$ (lowercase).

For any fixed $\delta_{\circ }$, the vertical line $\delta =\delta_{\circ }$ intersects all 480 curves, yielding a distribution of $Z_{s^{⊥}}$ values. Together with the probability mass function $m_{\delta_{\circ },\xi_{\circ }}$, this defines the sampling distribution of $Z_{s^{⊥}}$ when $\delta =\delta_{\circ }$ and $\xi_{\circ }=29$. Crucially, every such vertical distribution has mean zero and variance one, reflecting the standardization of the score.

The intersection of horizontal lines with $z_{s^{⊥}}$ provides confidence intervals through inversion. The lines z=±2 intersect the observed curve at points $\left(\delta_{lo},2\right)$ and $\left(\delta_{hi},-2\right)$, partitioning the parameter space into three regions:

- For $\delta \leq \delta_{lo}$: the observed $s^{⊥}$ exceeds 2 standard deviations above its expectation.
- For $\delta_{lo}<\delta <\delta_{hi}$: the observed $s^{⊥}$ lies within 2 standard deviations.
- For $\delta \geq \delta_{hi}$: the observed $s^{⊥}$ falls below −2 standard deviations.

The interval $\left(\delta_{lo},\delta_{hi}\right)$ forms an approximate 95% confidence interval for δ. The approximation quality depends on the normality of the vertical distributions, while the interval width depends on the slope of $z_{s^{⊥}}$—steeper slopes yield narrower intervals.

These calculations are conditional on ξ=29. Different nuisance parameter values yield different intervals, motivating our choice of the orthogonal parameterization (δ,ξ) where $\xi =n_{1}p_{1}+n_{2}p_{2}$. With this choice, the one-dimensional submanifolds $M_{\xi_{\circ }}=\xi^{-1}\left(\xi_{\circ }\right)$ and $M_{\delta }=\delta^{-1}\left(\delta \right)$ intersect transversally, and their tangent spaces are orthogonal at the intersection point.

Varying the horizontal line height provides confidence intervals at different levels. For all z≠0, these lines intersect each of the 480 curves, ensuring that confidence intervals exist for every sample point. The intersection of all confidence levels can be interpreted as a point estimate for δ. For sample points other than (0,0) and $\left(n_{1},n_{2}\right)$, this intersection equals the MLE—the point where $z_{s^{⊥}}$ crosses zero. At the boundary points (0,0) and $\left(n_{1},n_{2}\right)$, the curves never cross zero, yielding an empty intersection that corresponds to the nonexistence of the MLE.

The 2-standard deviation confidence interval (z=±2) for the log odds ratio δ is (−0.35, 2.27). The exact 95% confidence interval is (−0.40, 2.40) for nuisance parameter ξ equal to 29. This interval is a function of ξ. To obtain an interval that is the same for all values of the nuisance parameter, we take the union of intervals as ξ takes all values to obtain (−0.46, 2.42). The exact 95% confidence interval from Fisher’s exact test is (−0.57, 2.55).

### 5.2. Other Generalized Estimators

Point estimators naturally induce generalized estimators, though the relationship depends on the parameterization. For a parameterization θ and point estimator $\hat{\theta }$, if $\hat{\theta }\in H_{M}$, then the generalized estimator is $g_{\hat{\theta }}=\hat{\theta }-E\hat{\theta }$ when no nuisance parameters exist, or $g_{\hat{\theta }}={\left(\hat{\theta }-E\hat{\theta }\right)}^{⊥}$ with nuisance parameters present.

Consider the binomial family with proportion parameter *p*. The MLE $\hat{p}=y/n$ yields $g_{\hat{p}}=\hat{p}-p$, which is proportional to the score. However, for the log odds parameterization η=log(p/(1−p)), the MLE $\hat{\eta }=\log\left(\hat{p}/\left(1-\hat{p}\right)\right)$ satisfies $\hat{\eta }\left(0\right)=-\infty$ and $\hat{\eta }\left(n\right)=+\infty$, so $\hat{\eta }\notin H_{M}$. No generalized estimator exists for the unmodified log odds MLE.

A standard remedy adds a small constant ϵ to each cell, yielding the modified MLE:

$$\tilde{\eta }\left(y\right)=\log\left(\frac{y+\epsilon }{n-y+\epsilon }\right)\in H_{M}.$$

This modification ensures finite values throughout the sample space, enabling construction of the corresponding generalized estimator.

While the proportion MLE $\hat{p}$ could similarly be modified, this is rarely performed despite the MLE’s failure at the boundaries. The MLE’s parameter invariance allows its definition without reference to any specific parameterization: for y∉{0,n},

$${\hat{m}}_{y}=\arg\max_{m\in M}m\left(y\right).$$

This coordinate-free definition emphasizes the MLE’s geometric nature but obscures its boundary behavior.

For comparing two populations using log odds, the modified MLE yields the difference estimator $\hat{\delta }={\tilde{\eta }}_{1}-{\tilde{\eta }}_{2}$, with orthogonalized generalized estimator:

$$g=g_{\hat{\delta }}={\left(\hat{\delta }-E\hat{\delta }\right)}^{⊥}.$$

Like the orthogonalized score, *g* exhibits smoothness in parameters and distributional properties in the sample space: $g=g\left(y,\cdot \right)\in C^{1}\left(\Delta \right)$ for fixed *y*, and $g\left(\cdot ,\delta \right)\in H_{m}M$ for fixed δ. Both are orthogonal to the nuisance space. The critical distinction lies in their geometric location: while $s^{⊥}\in T\;M$, generally g∉TM unless $g=s^{⊥}$.

**Figure 2:**
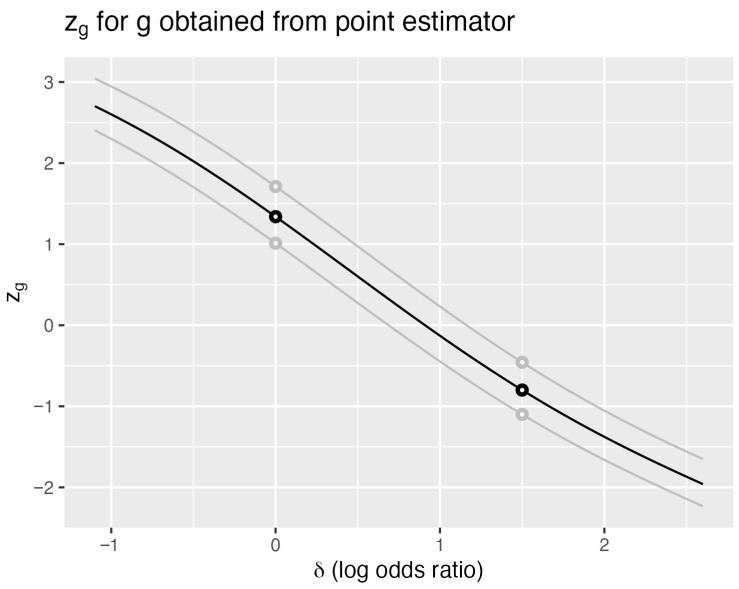
Cancer data—modified MLE estimator. Standardized generalized estimator $z_{g}$ based on the modified log odds difference $\hat{\delta }$, where 0.5 is added to each cell count. The observed data (18 of 23 surgery successes, 11 of 19 irradiation successes) yields the black curve. Two additional sample points are shown in gray. The nuisance parameter is fixed at ξ=29. Compare with Figure 1 to observe the flatter slope indicating lower Λ-information.

Figure 2 illustrates this distinction for the cancer data. The black curve shows $z_{g}$ for the observed sample with $\epsilon_{1}=\epsilon_{2}=0.5$ (adding 0.5 to each cell) and nuisance parameter $\xi_{\circ }=29$. Each of the 480 sample points generates a smooth curve, with two shown in gray. Vertical lines at any $\delta_{\circ }$ intersect these curves to yield distributions with mean zero and unit variance.

As with $z_{s^{⊥}}$, horizontal lines at $z=\pm z_{0}$ determine confidence intervals through their intersections with $z_{g}$. Steeper slopes produce narrower intervals, making the expected slope a natural efficiency measure. Differentiating the identity $EZ_{g}=0$ with respect to δ yields

$$E\frac{\partial Z_{g}}{\partial \delta }+E\left(Z_{g}Z_{s^{\;⊥}}\right)\sqrt{I_{\delta }^{⊥}}=0$$

Rearranging gives the fundamental inequality

(13) $${\left(E\frac{\partial Z_{g}}{\partial \delta }\right)}^{2}=\rho_{gs^{\;⊥}}^{2}I_{\delta }^{⊥}\leq I_{\delta }^{⊥}$$

where $\rho_{gs^{\;⊥}}$ is the correlation between *g* and $s^{⊥}$. Vos and Wu [12] define the left-hand side of (13) as the $\Lambda_{\delta }\left(g\right)$, Λ-information in *g* for parameter δ. The bound is attained only when $g=s^{⊥}$, establishing the optimality of the orthogonalized score:

(14) $$\Lambda_{\delta }\left(g\right)=\rho_{gs^{\;⊥}}^{2}I_{\delta }^{⊥}.$$

The square of the correlation is the same for any reparameterization of δ, so we can define the Λ-efficiency of *g* as

$${\mathrm{Eff}}^{\Lambda }\left(g\right)=\rho_{gs^{⊥}}^{2}.$$

Λ-efficiency is independent of the choice of interest or nuisance parameter. For example, Λ-efficiency will be the same whether we use the odds ratio or the log odds ratio. Λ-information, like Fisher information $I_{\delta }^{⊥}$, is a tensor.

The geometric interpretation is revealing: $\rho_{gs^{\;⊥}}$ measures the cosine of the angle between *g* and $s^{⊥}$ in $H_{m}M$. The information loss $\left(1-\rho_{gs^{\;⊥}}^{2}\right)I_{\delta }^{⊥}$ equals the squared sine of this angle times the total information. Estimators achieve full Λ-efficiency only when perfectly aligned with the orthogonalized score.

A crucial distinction emerges when testing $H_{0}:\delta =0$. Under this null hypothesis with simple random sampling, the standardized orthogonalized score becomes

$$Z_{s^{⊥}}=\frac{n_{2}^{1/2}Z_{1}-n_{1}^{1/2}Z_{2}}{\sqrt{n_{1}+n_{2}}}$$

which remains invariant across all parameterizations θ. This invariance reflects the geometric fact that $H_{0}:\delta =0$ is equivalent to $H_{0}:\theta_{1}=\theta_{2}$ regardless of the choice of θ.

In contrast, test statistics based on point estimators like ${\hat{\theta }}_{1}-{\hat{\theta }}_{2}$ depend critically on the parameterization. Tests based on proportions, log proportions, and log odds yield different statistics with different null distributions, even though they test the same hypothesis. The orthogonalized score provides a canonical, parameterization-invariant test that achieves maximum power against local alternatives.

The 2-standard deviation confidence interval (z=±2) for the log odds ratio δ is (−1.09, 2.95). The exact 95% confidence interval is (−0.43, 2.39) for nuisance parameter ξ equal to 29. The union of intervals over values for ξ is at least (−0.68, 2.47).

### 5.3. Discussion

Table 2 presents confidence intervals for the log odds difference δ computed using various methods. These intervals reveal substantial variation in both width and location, highlighting the importance of understanding the underlying geometric principles.

The orthogonalized score interval, whether computed at ξ=29 or maximized over all nuisance parameter values, falls within both the modified MLE and Fisher’s exact test intervals for this particular dataset. However, this nesting relationship is sample-specific and should not guide method selection. The choice among methods should depend on their theoretical properties rather than their behavior for any particular observed data.

The orthogonalized score offers three key advantages:

1. It attains the Fisher information bound, achieving maximum Λ-efficiency.
2. It requires no ad hoc modifications to handle boundary cases (unlike the MLE for log odds).
3. It provides parameterization-invariant inference for $H_{0}:\delta =0$, yielding identical test statistics whether we parameterize using proportions, log proportions, or log odds.

The R (version 4.4.1) package (version 4.4.1) exact2x2 (version 1.6.8) [14,15] implements several additional unconditional methods, each corresponding to different generalized estimators. While this diversity offers flexibility, it also highlights the need for principled comparison methods.

The geometric framework of generalized estimation provides this principled approach. By working in the Hilbert bundle, we obtain

- Unified treatment: Point estimators and test statistics become special cases of generalized estimators.
- Parameter invariance: Generalized estimators transform properly under reparameterization.
- Linear structure: The Hilbert bundle provides a natural vector space framework for combining and comparing estimators.
- Consistent comparison: Λ-information offers a single efficiency measure, replacing the multiple criteria (bias, variance, MSE) used for point estimators.

This geometric perspective reveals why the orthogonalized score achieves optimality: it lies in the tangent bundle TM, while other generalized estimators reside only in the larger Hilbert bundle HM. The information loss of any estimator equals $I_{\delta }^{⊥}$ times the squared sine of its angle from the tangent space—a geometric characterization that unifies and clarifies classical efficiency results.

## 6. Conclusions

This paper has demonstrated how the Hilbert bundle structure of statistical manifolds provides a unified geometric framework for statistical inference. By recognizing that points in a statistical manifold are probability distributions rather than abstract points, we extend the traditional tangent bundle framework to encompass a richer geometric structure that naturally accommodates both estimation and hypothesis testing.

The central insight is that generalized estimators—functions on the parameter space—serve as the fundamental inferential objects. The Λ-information of a generalized estimator *g* captures both its smooth structure across models in *M* and its distributional properties at each point. These dual aspects require different geometric descriptions: the smooth structure manifests through the graph of $Z_{g}$ in the (δ,z) plane, while the distributional properties are naturally characterized within the Hilbert bundle HM.

The information bound emerges as a geometric principle: the mean slope of $-Z_{g}$ equals $\sqrt{\Lambda (g)}$, and this slope is maximized precisely when *g* lies in the tangent bundle TM. Statistically, the bound is attained when $g=s^{⊥}$, the orthogonalized score. For any other generalized estimator, the information loss equals $\left(1-\rho_{gs^{⊥}}^{2}\right)I_{\delta }^{⊥}$, where $\rho_{gs^{⊥}}$ measures the correlation between *g* and $s^{⊥}$ as elements of HM. This correlation has a direct geometric interpretation: it equals the cosine of the angle between these functions in the Hilbert space.

The presence of nuisance parameters introduces an additional layer of geometric structure. Information loss due to nuisance parameters equals $\rho_{s\nu }^{2}I_{\delta }$, where $\rho_{s\nu }$ is the correlation between the score *s* and the nuisance direction $Z_{\nu }$. Crucially, this correlation—and hence the information loss—remains invariant under reparameterization of either interest or nuisance parameters. This invariance reflects a fundamental geometric fact: specifying a value $\delta_{\circ }$ for the interest parameter defines a submanifold $M_{\delta_{\circ }}=\delta^{-1}\left(\delta_{\circ }\right)$ rather than a single point in *M*. The increased inferential difficulty is precisely quantified by $\rho_{s\nu }^{2}$, the squared correlation between the score and the tangent space of $M_{\delta_{\circ }}$.

Our analysis of 2×2 contingency tables illustrates these principles concretely. The orthogonalized score achieves three key advantages over traditional approaches: it attains the information bound, requires no ad hoc modifications for boundary cases, and provides parameterization-invariant inference. The geometric framework explains why different confidence interval methods yield different results—they correspond to different generalized estimators with varying degrees of alignment with the tangent bundle.

This geometric perspective resolves longstanding tensions between estimation and testing frameworks. Rather than treating these as separate endeavors united only by computational tools like the likelihood function, we see them as complementary aspects of a single geometric structure. Point estimators, test statistics, and estimating equations all become special cases of generalized estimators, whose efficiency is uniformly measured by their Λ-information.

The Hilbert bundle framework thus provides both conceptual clarity and practical benefits. It reveals why certain statistical procedures are optimal, quantifies the cost of using suboptimal methods, and suggests principled ways to construct new inferential procedures. By shifting focus from points in parameter space to functions on the manifold, we gain a richer, more complete understanding of statistical evidence and its geometric foundations.

## Figures and Tables

**Table 1:** Fisher information per observation, $i_{\theta }$, for common parameterizations of the Bernoulli distribution expressed in the success parameter *p*.

Parameter θ	Information $i_{\theta }$ per Observation
$\log\left(\frac{p}{1-p}\right)$	p(1−p)
*p*	$\frac{1}{p(1-p)}$
logp	$\frac{1-p}{p}$

**Table 2:** Confidence intervals for the log odds difference δ using cancer treatment data. Computed using R version 4.4.1 with package exact2x2 version 1.6.8 [14]. All methods except Fisher’s exact test are unconditional; Fisher’s conditions on the observed total of 29 successes.

Method	R Function	95% Confidence Interval
Fisher-type adjusted	uncondExact2x2	(−∞, 0.57)
Simple asymptotic	uncondExact2x2	(−3.90, 2.48)
Score-based	uncondExact2x2	(−2.99, 0.43)
Orthogonalized score $s^{⊥}$	–	(−0.46, 2.42)
Fisher’s exact test	fisher.test	(−0.57, 2.55)

## Data Availability

No data created.
